# The impact of COVID-19 on quality of life among Lebanese adults: a cross-sectional study

**DOI:** 10.3389/fpubh.2025.1606720

**Published:** 2025-06-18

**Authors:** Samer A. Kharroubi, Ninette Geagea, Mona Zaidan

**Affiliations:** ^1^Department of Nutrition and Food Sciences, Faculty of Agricultural and Food Sciences, American University of Beirut, Beirut, Lebanon; ^2^School of Health and Related Research, The University of Sheffield, Sheffield, United Kingdom

**Keywords:** mental health, COVID-19, stress, anxiety, quality of life

## Abstract

**Background:**

Coronavirus disease 2019 (COVID-19) is an infectious disease caused by SARS-CoV-2. First identified in Wuhan, China, in December 2019, the virus rapidly spread worldwide, leading to its designation as a global pandemic. Beyond its significant mortality toll, concerns have emerged regarding its negative impact on the quality of life (QoL).

**Aims:**

This study aimed to estimate the prevalence of fear of COVID-19 and its impact on QoL among Lebanese adults and identify sociodemographic, behavioral, and health-related predictors influencing fear of COVID-19 and QoL during the pandemic.

**Methods:**

A cross-sectional online survey was conducted between October and December 2022 using a snowball sampling technique. A total of 402 respondents participated in the study. Statistical analyses, including multiple regression models, were conducted to determine predictors of fear and QoL deterioration.

**Results:**

The results demonstrated that 47% of participants experienced a negative impact on QoL, while 34% reported extreme fear of COVID-19. Key predictors of fear included education level (OR = 4.457, *p* = 0.028), number of household rooms (OR = 0.470, *p* = 0.048), and fear of limited access to treatment (OR = 0.865, *p* = 0.027). Factors associated with greater QoL deterioration included being female (OR = 2.239, *p* = 0.001), fear of limited access to treatment (OR = 3.032, *p* = 0.001), and having a worried family member (OR = 2.028, *p* = 0.016). Other significant predictors were household size, presence of mental illness, and emotional sharing with family or others. The study highlights the psychological and social burdens associated with COVID-19.

**Conclusion:**

Therefore, the findings highlight the urgent need to enhance access to healthcare, social support, and wellness programs to strengthen resilience in Lebanon. Enhancing access to healthcare, strengthening social support systems, and implementing wellness programs are crucial in fostering resilience in Lebanon. Addressing these issues can mitigate the psychological and social burdens of COVID-19, improving overall wellbeing.

## Introduction

Coronavirus disease 2019 (COVID-19) is an infectious disease caused by SARS-CoV-2, a virus that primarily attacks the human respiratory system (1). The most common symptoms are fever, cough, muscle aches, and dyspnea. Some unusual symptoms, such as vomiting and diarrhea, were recorded (2). Several studies exposed that person-to-person transmission is the most potential way for spreading COVID-19 infection (3, 4). It occurs primarily via direct contact or through sneezing/cough droplets spread by an infected individual (2). COVID-19 was first identified in Wuhan, Hubei province, China, in December 2019 and has spread rapidly to most major cities and towns in the world, leading the World Health Organization (WHO) to acknowledge this outbreak as a public health emergency of international concern (PHEIC) on 30 January 2020 (2) and issued considerations for the quarantine of people in the context of containment for COVID-19 on 29 February 2020. The guidelines defined who should be quarantined and the minimum duration of quarantine necessary to avoid the risk of additional transmission. Quarantine is the practice of isolating individuals (or populations) who have been exposed to an infectious disease. On the other hand, “isolation” refers to the separation of those who are known to be diseased (5). Although strict confinement and lockdown measures effectively reduced transmission, they also resulted in negative consequences.

In Lebanon, COVID-19 aggravated an already severe economic crisis. Since 2019, the country has been facing one of the world’s greatest economic crises. Four out of every ten Lebanese have no job, and half of the population live below the poverty line (6). Acute fuel shortages for both the private and public utilities have caused severe electricity blackouts across the country, with the public utility, Électricité du Liban (EDL), supplying as little as 2 h per day. In addition, medications have been in significant shortages with the health services being severely impacted. The situation worsened following the devastating 2020 Beirut port explosion (6). The country witnessed a dramatic increase in cases following the Beirut blast, reaching 680 daily cases by the end of August 2020. Every day in September, more than 1,000 cases were confirmed, exceeding the number of beds designated for the care of COVID-19 patients in many institutions. As the year’s conclusion drew near, illnesses spiraled out of control until they peaked in January 2021 with more than 6,000 daily cases (7). The first batch of COVID-19 vaccines arrived to Lebanon on 13 February 2021. The Ministry of Public Health (MOPH) effectively controlled the outbreak despite facing several political, financial, and economic obstacles (7).

Beyond the mounting death toll in numerous nations, concerns have been raised about the potential negative impact of the pandemic (and its mitigation strategies) on mental health and quality of life (QoL) (1). The psychological consequences of isolation and quarantine are complex, and they can have serious effects on people’s QoL. Not only did many individuals worry about physical symptoms linked to the infection, they also feared spreading the sickness to others. Adding to that, the fear caused significant irritation and disturbance caused by the loss of accustomed routines and activities. According to earlier studies, the longer the isolation time, the higher the incidence of poor mental health, post-traumatic stress disorder, and avoidance (4). Several studies have been conducted in Lebanon to examine the impact of COVID-19 on mental health. The Lebanese population experienced a variety of mental health disorders that have been brought on by the long-term traumas of conflict and domestic instability. The findings of a study by Salameh et al. (8) revealed that financial hardship and pandemic-related fears together further exacerbated stress and anxiety, going above and beyond the effects of each hardship alone. Moreover, according to a study by Grey et al. (9), 60% of people in self-isolation reported that their mental health depreciated since lockdown measures were imposed in Lebanon. In fact, early findings from an international survey of children and adults in 21 countries conducted in 2021 by United Nations International Children’s Emergency Fund (UNICEF) and Gallup revealed that an average of one in five young people aged 15–24 surveyed in Lebanon revealed that they often have little interest in doing things or feel depressed (10).

The consequences of COVID-19 continue to affect individuals and communities all over the world, especially in countries. In Lebanon, and several other countries, with minimal resources, recovery from COVID-19 has been restrained due to other factors and conditions. To date, the Lebanese population faces several economic and mental health problems, affecting the overall quality of life. Few studies have examined the impact of COVID-19 on quality of life (QoL) among students, and none of the studies have specifically investigated its effects on the general Lebanese adult population. Therefore, studying the impacts of COVID-19 on the quality of life among Lebanese adults is essential to address its ongoing effects and develop the necessary strategies and policies to obtain full recovery in all countries. Thus, this study aims to fill this gap by estimating the prevalence of fear of COVID-19 among Lebanese adults and identifying sociodemographic, behavioral, and health-related factors that may influence fear of COVID-19 and QoL during the pandemic.

## Materials and methods

### Study design and data source

This descriptive, cross-sectional study was conducted online between October and December 2022. Participants, over 18 years of age, were recruited using the snowball sampling technique, with the survey link distributed via a social media flyer (Appendix 1). The survey and study information were shared on various social media platforms, including Facebook pages and WhatsApp groups, inviting individuals to participate. The invitation included a link to the survey and an online consent form (Appendix 2), with the full survey provided in Appendix 3. The inclusion criteria were as follows: (1) willingness to participate, (2) individuals over 18 years of age with access to the internet, and (3) residing in Lebanon at the time of the survey. Participation was entirely voluntary and anonymous, with no penalties or consequences for non-participation. Participants were encouraged to ask questions or seek clarification before providing consent. Ethics approval for the study was obtained from the Institutional Review Board (IRB) at the American University of Beirut (AUB).

### Survey format

The survey was structured into multiple sections:

1. *Sociodemographic Characteristics*—This section collected data on participants’ age, gender, marital status, education level, occupation, income, nationality, region, living conditions, and current household monthly income.
2. *Health-Related* Var*iables*—Participants were asked about the presence of family members, friends, or colleagues who had contracted COVID-19.
3. *Behavioral Factors*—This section included questions related to smoking and alcohol consumption.
4. *Fear of COVID-19 and Quality of Life*—This section assessed participants’ levels of fear and the perceived impact of COVID-19 on their quality of life (QoL).

### Social and family support

Participants also completed a set of five questions evaluating social and family support, including support from friends, support from family members, sharing feelings with family, sharing feelings with others, and caring about family members’ emotions (11, 12). Response options included “decreased,” “same as before,” and “increased.”

### Fear of COVID-19 scale

The Fear of COVID-19 Scale is a 7-item tool designed to measure the extent of fear of getting or thinking about the disease in adults. It was validated by Ahorsu et al. and supported by two scales, namely, the Hospital Anxiety and Depression Scale and the Perceived Vulnerability to Disease Scale. The items include being afraid of COVID-19, uncomfortable thinking about COVID-19, hands becoming clammy when thinking about COVID-19, being afraid of losing life, becoming nervous or anxious when watching news, worrying and being unable to sleep, and having an increased heart rate when thinking about COVID-19. Each item is rated on a five-point Likert scale from 1 (strongly disagree) to 5 (strongly agree), with total scores ranging from 7 to 35. Higher scores indicate greater fear of COVID-19. Participants with scores ≥17.5 were categorized as experiencing extreme fear, while those scoring below this threshold were classified as having normal fear (13).

### COVID-19 impact on quality of life (COV19-QoL) scale

The COV19-QoL scale consists of six items, each rated on a five-point scale (1 = completely agree to 5 = completely disagree). Higher scores indicate a greater perceived impact of COVID-19 on QoL. Scores were analyzed per item or as an overall measure. To generate a total score, responses were summed and divided by the number of items (6), yielding an average score. This average can then be compared to the theoretical midpoint of the scale (3 on a 5-point scale) to assess the level of QoL impact (14).

### Statistical analysis

Data from LimeSurvey were generated and collected on Excel sheets and then transferred to IBM SPSS® software version 23.0 for further analysis. After that, computations of the different scores were completed to categorize participants based on respective cutoffs. For descriptive analysis, frequencies and percentages were reported for all categorical variables. Bivariate analysis was performed using simple logistic regressions to examine associations between independent variables (sociodemographic, behavioral, and health-related factors) and each dependent variable (fear of COVID-19 and quality of life). Significant associations (*p*-value < 0.05) were further analyzed using multiple logistic regression models to adjust for confounding factors. Adjusted odds ratios (AORs) with corresponding confidence intervals were reported for significant predictors.

## Results

### Sociodemographic characteristics

Our survey received 739 responses, out of which 402 were full responses. Only complete responses were included in the analysis (*N* = 402). Table 1 shows the sociodemographic characteristics of the participants. More than half of the participants (62.2%) were females. Younger adults represented the largest proportion of the sample population, with 34% aged 18–24 and 25.1% aged 25–29. Moreover, 61.5% of the participants were single, and the majority (79.5%) held a university degree. With regard to employment status, 44% of the studied population were full-time employees, 12.9% were self-employed, 13.9% were part-time employed, 19.7% were unemployed, and 6.8% were actively seeking employment. Nearly two-third of the participants stated residing in Mount Lebanon (64.5%).

**Table 1:** Sociodemographic characteristics of participants in the study sample (*N* = 402).

Characteristics		*n* (%)
Gender	Male	125 (31.1)
	Female	250 (62.2)
	Prefer not to answer	27 (6.7)
Age	18–24	122 (34.0)
	25–29	90 (25.1)
	30–39	74 (20.6)
	40–49	38 (10.6)
	≥50	35 (9.7)
Marital status	Married	121 (31.7)
	Single	235 (61.5)
	Engaged	19 (5)
	Divorced/widowed/separated	7 (1.8)
Education level	Pre-high school	20 (5.2)
	High school	59 (15.3)
	University undergraduate (BS, BA, technical, vocational, etc.)	142 (36.8)
	University graduate (MS, MBA, PhD, MD, etc.)	165 (42.7)
Dwelling region	Beirut	79 (20.5)
	Mount Lebanon	249 (64.5)
	South Lebanon	29 (7.5)
	Bekaa	5 (1.3)
	North Lebanon	24 (6.2)
Employment type	Self-employed	49 (12.9)
	Full-time employee	167 (44)
	Part-time employee/daily laborer	53 (13.9)
	Unemployed, not seeking employment (student, housewife, handicapped, retired, etc.)	75 (19.7)
	Unemployed, actively seeking employment	26 (6.8)
	Other	10 (2.7)

### Fear of COVID-19 and negative impact of COVID-19 on QoL

Table 2 presents the prevalence of fear of COVID-19 and its negative impact on quality of life (QoL). Based on the Fear of COVID-19 Scale, 129 participants (34.4%) were classified as experiencing extreme fear of COVID-19 and its complications. Regarding the impact of COVID-19 on QoL, nearly half of the participants (47.1%) scored ≥3, indicating a high negative impact on their quality of life.

**Table 2:** Prevalence of fear of COVID-19 and negative impact of COVID-19 on QoL.

Characteristics		*n* (%)
Fear of COVID-19	Normal fear of COVID-19	246 (65.6)
	Extreme fear of COVID-19	129 (34.4)
Impact of COVID-19 on QoL	Low impact on quality of life	190 (52.9)
	Higher impact on quality of life	169 (47.1)

### Simple and multiple logistic regression analyses

#### Factors associated with fear of COVID-19

Several factors were significantly associated with fear of COVID-19 among participants. As shown in Table 3, education level emerged as a key predictor, with high school students being more likely to experience fear of COVID-19 compared to pre-school students (OR = 4.457, *p* = 0.028). Other significant predictors comprised the number of rooms in the household and fear of limited access to treatment. More specifically, participants living in homes with ≥7 rooms were less likely to experience fear of COVID-19 compared to those with <5 rooms (OR = 0.470, *p* = 0.048). Moreover, individuals concerned about access to treatment had significantly higher odds of experiencing fear of COVID-19 compared to those without such concerns (OR = 1.865, *p* = 0.027, Table 4).

**Table 3:** Associations of sociodemographic variables with fear of COVID-19 and the impact of COVID-19 on quality of life.

Sociodemographic variables	Fear of COVID-19		Impact of COVID-19 on the quality of life	
	Simple regression OR (95% CI); *p*-value	Multiple regression OR (95% CI); *p*-value	Simple regression OR (95% CI); *p*-value	Multiple regression OR (95% CI); *p*-value
Gender				
Male				
Female	1.378 (0.856–2.219); 0.186		**2.276 (1.418–3.651); 0.001**	**2.239 (1.386–3.618); 0.001**
Prefer not to answer	1.868 (0.749–4.658); 0.180		**2.928 (1.148–7.471); 0.025**	5.348 (1.265–22.607); 0.23
Age				
18–24				
25–29	1.251 (0.679–2.305); 0.472		0.864 (0.471–2.028); 0.615	
30–39	1.600 (0.843–3.034); 0.150		0.886 (0.233–1.344); 0.696	
40–49	2.016 (0.921–4.410); 0.079		0.351 (0.127–1.304); 0.351	
> = 50	**2.867 (1.217–6.463); 0.011**	0.906 (0.295–2.776); 0.862	1.000 (0.230–2.499); 1.000	
Marital status				
Married	**2.101 (1.302–3.389); 0.002**	1.924 (0.302–12.261); 0.489	2.298 (0.870–6.068); 0.093	
Single				
Engaged	2.538 (0.965–6.680); 0.059		1.472 (0.923–2.347); 0.104	
Divorced/widowed/separated	1.015 (0.192–5.366); 0.986		1.788 (0.390–8.184); 0.454	
Education level				
Pre-high school				
High school	**0.138 (0.039–0.484); 0.002**	**4.457 (1.179–16.856); 0.028**	1.972 (0.605–6.433); 0.260	
University undergraduate (BS, BA, technical, vocational, etc.)	**0.182 (0.056–0.588); 0.004**	1.541 (0.680–3.492); 0.300	2.375 (0.782–7.215); 0.127	
University graduate (MS, MBA, PhD, MD, etc.)	**0.125 (0.039–0404); 0.001**	1.531 (0.879–2.667); 0.133	1.738 (0.575–5.258); 0.328	
Primary nationality				
Lebanese				
Prefer not to answer	0.631 (0.242–1.641); 0.345		1.146 (0.071–18.475); 0.924	
Other	1.250 (0.067–23.259); 0.881		1.432 (0.551–3.722); 0.461	
Employment type				
Self-employed				
Full-time employee	1.607 (0.785–3.291); 0.194		1.740 (0.864–3.504); 0.121	
Part-time employee/daily laborer	1.121 (0.465–2.702); 0.799		1.243 (0.526–2.936); 0.620	
Unemployed, not seeking employment (student, housewife, handicapped, retired, etc.)	1.255 (0.562–2.803); 0.579		2.235 (1.022–4.889); 0.044	
Unemployed, actively seeking employment	0.964 (0.328–2.828); 0.946		2.285 (0.827–6.314); 0.111	
Other	**7.846 (1.400–43.958); 0.019**	0.200 (0.027–1.510); 0.119	3.222 (0.676–15.352); 0.142	
Dwelling region				
Beirut				
Mount Lebanon	1.217 (0.694–2.134); 0.492		0.968 (0.561–1.671); 0.907	
South Lebanon	1.208 (0.483–3.022); 0.687		0.667 (0.272–1.635); 0.376	
Bekaa	–		0.687 (0.108–4.378); 0.691	
North Lebanon	1.159(0.431–3.120); 0.770		0.589 (0.218–1.588); 0.295	
Income status				
No income				
Low <675,000 LBP (450USD)	1.182 (0.474–2.944); 0.720		0.934 (0.293–2.977); 0.497	
Moderate 675,000 - 1,500,000 LBP (450–1,000 USD)	1.262 (0.597–2.665); 0.543		0.507 (0.171–1.502); 0.126	
Intermediate 1,500,000-3,000,000 LBP (1,000–2,000 USD)	1.378 (0.668–2.841); 0.385		0.583 (0.209–1.628); 0.105	
High > 3,000,000 LBP (2,000 USD)	1.647 (0.820–3.307); 0.161		0.718 (0.252–2.047); 0.323	
Prefer not to answer	0.988 (0.328–2.974); 0.983		0.282 (0.074–1.072); 0.066	
Household size				
<4 persons				
4 persons	1.018 (0.580–1.786); 0.952		1.394 (0.735–2.643); 0.208	
5 persons	1.208 (0.667–2.187); 0.533		1.063 (0.519–2.177); 0.741	
≥ 6 persons	1.130 (0.548–2.329); 0.740		0.750 (0.314–1.795); 0.335	
Number of rooms				
<5 rooms				
5 rooms	0.783 (0.444–1.380); 0.398		1.167 (0.661–2.058); 0.594	1.191 (0.667–2.125); 0.554
6 rooms	1.005 (0.547–1.850); 0.986		1.110 (0.614–2.005); 0.730	0.986 (0.537–1.811); 0.964
≥7 rooms	**0.312 (0.155–0.629); 0.001**	**0.470 (0.222–0.995); 0.048**	**0.484 (0.261–0.899); 0.022**	**0.482 (0.355–0.910); 0.024**

Estimates shown in bold are those that are statistically significant at *p* < 0.05.

**Table 4:** Associations of health-related variables with the fear of COVID-19 and the impact of COVID-19 on the quality of life.

Health-related variables	Fear of COVID-19		Impact of COVID-19 on the quality of life	
	Simple regression OR (95% CI); *p*-value	Multiple regression OR (95% CI); *p*-value	Simple regression OR (95% CI); *p*-value	Multiple regression OR (95% CI); *p*-value
Alcohol consumption				
Previous				
None	1.954 (0.284–13.436); 0.496		1.437 (0.289–7.152); 0.658	
Occasional	1.200 (0.176–8.197); 0.852		0.621 (0.130–2.975); 0.551	
Regular	0.914 (0.111–7.506); 0.934		0.399 (0.70–2.277); 0.301	
Cigarette smoking				
Previous				
None	0.627 (0.70–2.117); 0.452		0.564 (0.087–3.657); 0.548	
Occasional	0.510 (0.066–1.901); 0.316		0.784 (0.107–5.772); 0.811	
Regular	0.655 (0.133–2.370); 0.519		1.021 (0.251–4.146); 0.977	
Waterpipe smoking				
Previous				
None	3.178 (0.378–26.743); 0.287		2.960 (0.181–48.426); 0.447	
Occasional	4.737 (0.513–43.728); 0.170		4.583 (0.237–88.602); 0.314	
Regular	1.412 (0.131–15.266); 0.776		4.041 (0.192–85.162); 0.369	
Violence at home				
Physical/verbal/other violence				
No violence	1.268 (0.677–2.377); 0.459		**0.363 (0.192–0.686); 0.002**	0.690(0.327–1.450); 0.331
Current health coverage				
No health coverage				
Private insurance	1.058 (0.457–2.343); 0.856		1.078 (0.474–2.452); 0.857	
Social security	1.332 (0.484–2.896); 0.422		1.267 (0.522–3.077); 0.601	
Other public coverage	2.100 (0.700–7.797); 0.106		0.522 (0.153–1.776); 0.298	
Mental illness				
Do you have a mental illness?				
Yes				
No	0.625 (0.248–1.571); 0.317		**0.343 (0.175–0.671); 0.002**	**0.398 (0.170–0.931); 0.034**
Prefer not to answer	0.519 (0.069–3.878); 0.523		1.581 (0.291–8.596); 0.596	1.299 (0.190–8.892); 0.790
Do you have a friend with a mental illness?				
Yes				
No	1.110 (0.547–2.252); 0.772		**0.492 (0.304–0.797); 0.004**	0.856 (0.437–1.679); 0.652
Prefer not to answer	1.060 (0.205–5.483); 0.944		0.684 (0.237–1.977); 0.483	0.608 (0.150–2.464); 0.486
Do you have a family member diagnosed with a mental illness?				
Yes				
No	1.360 (0.654–2.826); 0.410		**0.586 (0.350–0.983); 0.043**	1.173 (0.600–2.292); 0.641
Prefer not to answer	6.549 (0.855–50.184); 0.071		0.930 (0.231–3.743); 0.919	0.751 (0.108–5.202); 0.772
Chronic illness				
Do you have a chronic illness?				
Yes				
No	1.465 (0.379–5.665); 0.580		**0.398 (0.216–0.735); 0.003**	0.888 (0.279–2.825); 0.840
Prefer not to answer	–		1.029(0.87–12.122); 0.982	–
Treatment for chronic illness				
Regular treatment				
No regular treatment	0.814 (0.194–3.414); 0.779		**0.438 (0.208–0.922); 0.030**	0.686 (0.199–2.365); 0.550
N/A	0.355 (0.075–1.681); 0.192		**0.371 (0.189–0.729); 0.004**	0.561 (0.150–2.106); 0.392
Fear no access to treatment				
No				
Yes	**1.718 (1.013–2.915); 0.045**	**1.865 (1.072–3.243); 0.027**	**4.038 (2.301–7.089); 0.000**	**3.032 (1.578–5.828); 0.001**
N/A	0.818 (0.481–1.391); 0.936		1.352 (0.668–2.736); 0.402	1.515 (0.803–2.858); 0.200
Do you have a family member diagnosed with a chronic illness?				
No				
Yes	0.984 (0.275–1.531); 0.942		**2.332 (1.496–3.636); 0.000**	1.447 (0.843–2.483); 0.180
N/A	0.459 (0.057–1.286); 0.138		0.239 (0.051–1.125); 0.217	0.436 (0.124–1.525); 0.194
Worried family member				
No				
Yes	1.519 (1.405–2.394); 0.072		**3.214 (2.031–5.086); 0.000**	**2.028 (1.144–3.595); 0.016**
N/A	0.898 (0.329–2.450); 0.834		1.375 (0.320–5.912); 0.789	1.095 (0.307–3.902); 0.889

Estimates shown in bold are those that are statistically significant at *p* < 0.05.

### Factors associated with the impact of COVID-19 on the QoL

Table 3 presents the sociodemographic factors influencing the impact of COVID-19 on quality of life. Key predictors include gender, number of rooms in the household, mental illness, fear of no access to treatment, and worried family members. In particular, the impact of COVID-19 was significantly higher among women than among men (OR = 2.239, *p* = 0.001). Participants living in households with ≥7 rooms experienced a lower impact on QoL compared to those with <5 rooms (OR = 0.482, *p* = 0.024), whereas individuals without mental illness had lower odds of experiencing a higher impact on QoL compared to those with mental illness (OR = 0.398, *p* = 0.034). Furthermore, participants concerned about treatment access were three times more likely to experience a higher negative impact on QoL compared to those who were not concerned (OR = 3.032, *p* = 0.001). Participants with a worried family member were twice as likely to report a higher impact of COVID-19 on their QoL compared to those without such concerns (OR = 2.028, *p* = 0.016, Table 4). In addition, participants who reported that sharing feelings with family was the same as before had 70% lower odds of experiencing a higher QoL impact compared to those who reported decreased sharing (OR = 0.308, *p* = 0.005), and those who reported increased sharing of feelings with family had 62% lower odds of a higher impact (OR = 0.385, *p* = 0.034). Finally, participants who reported no change in sharing feelings with others during difficult times had 54% lower odds of experiencing a higher QoL impact compared to those with decreased sharing (OR = 0.464, *p* = 0.028, Table 5).

**Table 5:** Associations of social variables with the fear of COVID-19 and the impact of COVID-19 on the quality of life.

Social variables	Fear of COVID-19		Impact of COVID-19 on the quality of life	
	Simple regression OR (95% CI); *p*-value	Multiple regression OR (95% CI); *p*-value	Simple regression OR (95% CI); *p*-value	Multiple regression OR (95% CI); *p*-value
Getting support from friends				
Decreased				
Same as before	0.640 (0.391–1.048); 0.076		**0.514 (0.318–0.831); 0.007**	0.788 (0.45–1.376); 0.402
Increased	1.432 (0.762–2.691); 0.265		0.767 (0.350–1.680); 0.190	0.795 (0.365–1.729); 0.562
Getting support from family				
Decreased				
Same as before	0.965 (0.488–1.911); 0.920		**0.399 (0.198–0.803); 0.010**	0.751 (0.336–1.677); 0.484
Increased	1.506 (0.736–3.081); 0.262		**0.356 (0.170–0.746); 0.006**	0.556 (0.217–1.426); 0.222
Sharing feelings with family				
Decreased				
Same as before	**0.420 (0.218–0.810); 0.010**		**0.175 (0.084–0.362); 0.000**	**0.308 (0.135–0.705); 0.005**
Increased	0.859 (0.455–1.622); 0.639	0.533 (0.252–1.131); 0.101	**0.266 (0.129–0.574); 0.000**	**0.385 (0.159–0.932); 0.034**
Sharing feelings with others when in blue				
Decreased				
Same as before	**0.526 (0.296–0.937); 0.029**		**0.256 (0.143–0.458); 0.000**	**0.464 (0.234–0.922); 0.028**
Increased	1.314 (0.730–2.365); 0.363	0.650 (0.334–1.265); 0.204	0.696 (0.380–1.277); 0.242	1.248(0.601–2.593); 0.552
Caring with family members’ feelings				
Decreased				
Same as before	0.613 (0.350–2.808); 0.314		1.799 (0.592–5.471); 0.259	
Increased	1.051 (0.410–3.380); 0.915		2.005 (0.650–6.178); 0.435	

Estimates shown in bold are those that are statistically significant at *p* < 0.05.

## Discussion

Quality of life is a multifaceted concept that encompasses an individual’s overall wellbeing across various domains, including physical, psychological, social, and environmental aspects. To our knowledge, this is the first study to assess fear levels and quality of life among the general adult population in Lebanon during the COVID-19 pandemic. Several factors were found to be significantly associated with fear of COVID-19 and its impact on quality of life.

Nearly one-third of the population (34.4%) reported extreme fear of COVID-19 compared to 65.6% who reported normal fear of COVID-19, which is quite higher than the number reported in a study conducted in Europe, where 18.1% exhibited strong fear of COVID-19 (15). Our findings indicate that high school graduates were more likely to exhibit fear of COVID-19 than pre-high school participants. Contrary to our findings, many studies reported that individuals with a higher education level may utilize more effective coping mechanisms, resulting in lower stress and fear levels being reported (16). While our study focused on assessing fear of COVID-19, a related study conducted in Turkey examined other psychological outcomes, including perceived stress and hopelessness levels. The study found no significant association between education level and perceived stress and hopelessness levels (17). Our results could have been influenced by high school graduates’ access to news and social media, where reports about COVID-19 were widespread. In addition, housing conditions turned out to affect fear levels with participants who have 7 rooms at home were less likely to be afraid of COVID-19 compared to those who have <5 rooms. Knowing that the housing conditions in Lebanon vary according to regional differences and the economic status of the individuals, and the average Lebanese households size, as defined by the Labor Force and Household Living Conditions Survey conducted by Lebanon’s Central Administration for Statistics (CAS) in 2018–2019, is 3.8 (18), the increased number of rooms provides excess space for self-isolation and quarantine, so family members of the same household may have a minimal fear of being infected with COVID-19 when another member is feeling ill. Having no access to treatment resulted in greater fear, suggesting that the availability and accessibility of treatment play a significant role in the level of fear and anxiety about the COVID-19 pandemic especially during the Lebanese economic crisis which caused major shortages in medications.

In addition, almost half of the participants reported a higher impact of the COVID-19 on the quality of life (47.1%), compared to 52.9% who reported a lower impact on the quality of life. The results were slightly lower than those of a study that was performed in Greece, in which the quality of life was worsened in 57% of the participants (19). Factors associated with the impact of COVID-19 on the quality of life include gender. The impact of COVID-19 on the quality of life was higher among females than males. Studies have shown that women experienced higher levels of anxiety, depression, and stress during the pandemic. A comprehensive study across 59 countries found that women reported greater trauma-related distress and had more difficulty decompressing compared to men. In addition, women exhibited decreased frustration tolerance and poorer sleep quality, leading to an increased likelihood of using sleep medications or natural remedies (20). Given that elevated stress levels negatively impact psychological wellbeing and, in turn, quality of life, our findings align with a study conducted among nursing students in Turkey, where female participants reported higher perceived stress levels than their male counterparts (21). However, another Turkish study found no significant association between gender and levels of perceived stress or hopelessness related to COVID-19 (17). These contrasting results may reflect sociocultural differences between Lebanon and Turkey, including the varying roles of women in each society. Furthermore, Lebanon’s compounded crises—such as the ongoing economic collapse, political instability, and the aftermath of the Beirut port explosion—may have amplified the gender disparities observed in our study. Moreover, participants living in homes with 7 or more rooms were less likely to experience a negative impact of COVID-19 on their quality of life compared to those in homes with fewer than 5 rooms. Larger homes provide more space for family members to maintain physical distance and allow individuals to maintain privacy and have improved home environment for remote work and education. A study among university students in Italy suggests that smaller living spaces may exacerbate negative mental health and QoL outcomes during lockdown (22). Furthermore, sharing feelings with family members and with others when in blue resulted in lower impacts of COVID-19 on the quality of life. These findings were found comparable with another study done in the MENA region, where more than half of the respondents indicated receiving more support from their family members and being more attentive to their family members’ emotions during the pandemic (23). These favorable effects on mental wellbeing might have assisted participants in dealing with the pandemic’s impact on quality of life (23). Adding to that, having a higher impact of COVID-19 on quality of life is less in individuals with no mental illness as compared to individuals with mental illness. The findings are in line with the key findings of the WHO’s scientific brief of mental health 2022, and individuals with pre-existing mental conditions are at an increased likelihood of experiencing severe illness and mortality from COVID-19 and, as such, should be recognized as a high-risk population when diagnosed with infection. Moreover, having no access to treatment or having a worried family member increases the impact on the participant’s quality of life. These findings were similar to the results obtained by Salameh et al. (8), where the fear of having no access to treatment and having a worried family member were found to correlate with stress and anxiety amid COVID-19 pandemic thus affecting the quality of life.

Several limitations may have influenced our findings. First, it is well established that in studies involving volunteers, individuals possessing the characteristic under investigation may be less inclined to participate compared to those who do not, potentially resulting in selection bias (24). Moreover, the use of snowball sampling may have further contributed to selection bias by overrepresenting certain groups while underrepresenting others. Second, the study relied on self-reported data, which may be subject to social desirability bias. Third, the study employed a primarily quantitative approach. Future research incorporating qualitative methods could offer a more comprehensive understanding of the impact of COVID-19 on QoL among the Lebanese population and complement the findings of quantitative analyses. Despite these limitations, our study provides valuable insights that can support the development of targeted interventions aimed at improving health-related quality of life (HRQoL) in the general Lebanese population.

## Conclusion

The results of this study indicate that the COVID-19 pandemic was significantly associated with mental health outcomes among Lebanese adults. Therefore, the lasting and prolonged effects of the COVID-19 pandemic, worldwide, need to be fully recovered. Consequently, it is recommended that the Lebanese government and policymakers design and implement targeted psychological support programs for adults to promote their mental health and overall wellbeing. In addition, raising awareness, among the adult population, about COVID-19 and other viruses to understand the viruses and their transmission routes is crucial to prevent fear and avoid the impacts of these viruses on the quality of life of individuals. Moreover, the media should be monitored in such cases to avoid the information provided. The findings also underscore the need to enhance access to treatment, social support, and wellness programs in order to strengthen resilience to future crises and improve the mental health outcomes of the Lebanese population.

## Data Availability

The raw data supporting the conclusions of this article will be made available by the authors, without undue reservation.
