# Supplementary material for: The impact of COVID-19 on quality of life among Lebanese adults: a cross-sectional study
Source: Front Public Health. 2025 Jun 18;13:1606720. doi: 10.3389/fpubh.2025.1606720 (PMC12213388; doi:10.3389/fpubh.2025.1606720)
Supplement: Supplementary file 1 [file Data_Sheet_1.pdf]

Appendix 1-Social Media Announcement (English)

The Impact of COVID-19 on Mental health and Quality of Life Amongst Lebanese Adults: Cross-sectional Study

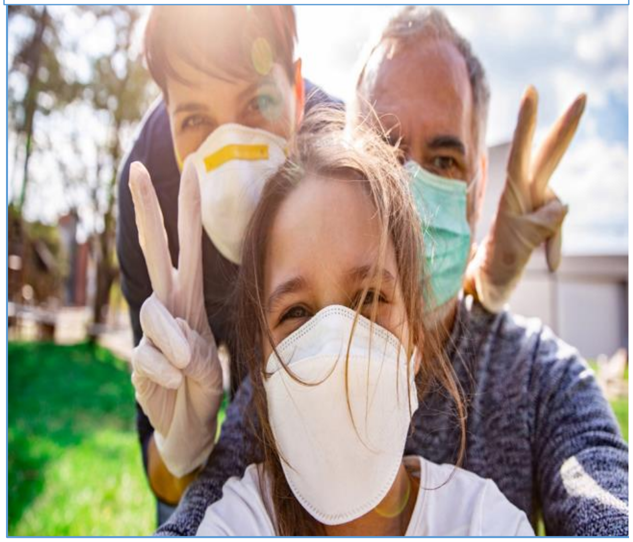

Dr. Samer Kharroubi

American University of Beirut

Approved by the AUB-IRB

You are invited to take part in a research study that aims to explore the impact of COVID-19 on depression, anxiety, stress and quality of life of Lebanese adults.

If interested, you can complete a short online survey that will not take more than 30 minutes of your time.

For more information, please email Dr. Samer A Kharroubi: *sk157@aub.edu.lb.*
The invitation is not an official communication from AUB/AUBMC

Appendix 1-Social Media Announcement (Arabic)

تأثير جائحة كورونا (COVID-19) على الصحة النفسية وجودة الحياة بين اللبنانيين: دراسة مقطعية

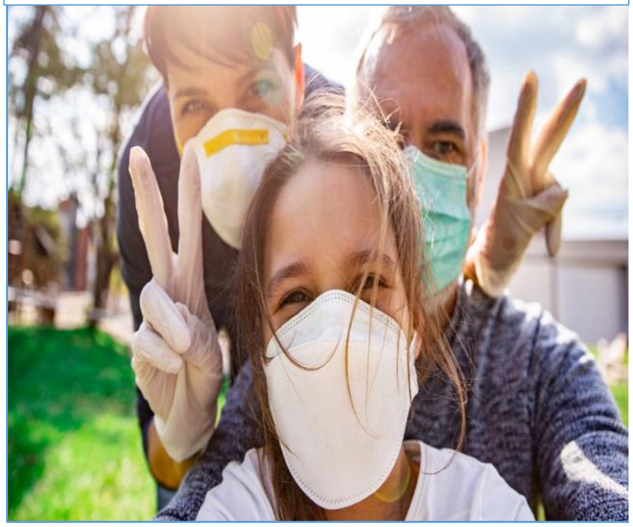

الدكتور سامر خَرّوبي

الجامعة الأمريكية في بيروت

تمت الموافقة عليها من قبل AUB-IRB

أنت مدعو/ة للمشاركة في دراسة بحثية حول تأثير جائحة كورونا (COVID-19) على الصحة النفسية
الاكتئاب والقلق والتوتر ونوعية حياة اللبنانيين.

للراغبين ، يمكنك إكمال استطلاع قصير عبر الإنترنت
لن يستغرق أكثر من 30 دقيقة من وقتك.

لمزيد من المعلومات ، يرجى إرسال بريد إلكتروني إلى sk157@aub.edu.lb.

الدعوة ليست رسالة رسمية من الجامعة الأميركية في بيروت / المركز الطبي في الجامعة الأميركية في بيروت
