# Supplementary material for: The impact of COVID-19 on quality of life among Lebanese adults: a cross-sectional study
Source: Front Public Health. 2025 Jun 18;13:1606720. doi: 10.3389/fpubh.2025.1606720 (PMC12213388; doi:10.3389/fpubh.2025.1606720)
Supplement: Supplementary file 2 [file Data_Sheet_2.pdf]

Appendix 2- Online Consent (English)

<u>Consent to participate in an Online Research Study</u>

This notice is for an AUB-IRB Approved Research Study for **Dr. Samer Kharroubi** at AUB.

It is not an Official Message from AUB

You are invited to participate in a research study entitled **The Impact of COVID-19 on Mental health and Quality of Life Amongst Lebanese Adults: Cross-sectional Study** conducted by **Dr. Samer Kharroubi**, Faculty of **Agricultural and Food Sciences** at the **American University of Beirut**. The conduct of this study will adhere to the IRB approved protocol.
The IRB approved method for approaching subjects is via an online survey link that will be distributed to all participants using the snowball sampling technique. The survey link and information will be posted on different social media platforms including Facebook Pages and WhatsApp groups, where participants will be invited to the research. The invitation will include the link to the survey and consent form. The purpose of the present study is to explore the impact of the COVID-19 on the mental health and quality of life (QoL) of the Lebanese adult population.

PROCEDURES

This message invites you to read the consent document and to consider whether you want to be involved in the study. It also urges you note the following:

- Participation is completely voluntary.
- Completing the questionnaire will take around **20** to **30 minutes**.
- Only the data you provide in the questionnaire will be collected and analyzed.
- The research team will not have access to your name or contact details.
- The results of the survey will be published in research articles /thesis/project report available in printed from and electronically from AUB Libraries.
- The inclusion criteria will be (1) willingness to participate, (2) individuals over 18 years of age with access to the internet, and (3) residing in Lebanon at the time of the survey.

POTENTIAL BENEFITS TO SUBJECTS AND/OR TO SOCIETY

You will not receive payment for participation in this study.

The results of the study will help to identify potentially vulnerable groups or modifiable factors to design and provide specific psychological promotion programmes for adults with the aim of promoting their mental health and wellbeing during the COVID-19 era.

POTENTIAL RISKS TO SUBJECTS AND/OR SOCIETY

The risks of the study are minimal. But due to dealing with potentially sensitive questions regarding mental health and fear of COVID-19, there is a potential concern of triggering negative emotions,

evoking memories that you may find stressful or upsetting. In such a case, you may receive professional support at your own expense by contacting:

- During working hours (8 am – 5 pm): contacting the outpatient services of the Department of Psychiatry at the American University of Beirut Medical Center (01-759620;01-350000, Ext. 5650) stating you have taken part in the survey and requesting a consultation OR asking to speak to the nurses.

CONFIDENTIALITY

The collected data will remain confidential and anonymous.

Records will be monitored and may be audited by the IRB while assuring confidentiality.

PARTICIPATION AND WITHDRAWAL

If you voluntarily consent to take part in this study, you can change your mind and withdraw at any time without consequences of any kind. Refusal to participate or withdrawal from the study will involve no penalty or loss of benefits to which the subject is otherwise entitled, and neither will it affect their relationship with their organization and AUB/AUBMC.

QUESTIONS ABOUT THE STUDY

If you have any questions about the study, can contact the research team at **Dr. Samer A Kharroubi, Faculty of Agricultural & Food Sciences-AUB; Tel: 961-1-350000 (Ext 4541) E-mail: sk157@aub.edu.lb**

ACCESS TO THE SURVEY

If after reading the consent document and having you questions answered, you voluntarily agree to take part in the study; you can access the survey by clicking on the following link.

CONCERNS OR QUESTIONS ABOUT YOUR RIGHTS

If you have concerns about the study or questions about your rights as a participant, you can contact the **AUB IRB Office at: Social & Behavioral Sciences Institutional Review Board [01-350000, Extension: 5445, irb@aub.edu.lb].**

Appendix 2- Online Consent (Arabic)

<u>الموافقة على المشاركة في دراسة بحثية عبر الإنترنت</u>

هذا الإشعار خاص بدراسة بحثية معتمدة من الجامعة الأميركية في بيروت ومجلس المراجعة المؤسسية للدكتور سامر الخروبي في الجامعة الأميركية في بيروت.

ليست رسالة رسمية من الجامعة الأمريكية في بيروت

أنت مدعو للمشاركة في دراسة بحثية بعنوان " تأثير جائحة كورونا (COVID-19) على الصحة النفسية وجودة الحياة بين اللبنانيين: دراسة مقطعية "أجراها الدكتور سامر خروبي ، كلية الزراعة وعلوم الأغذية في أمريكا .جامعة بيروت .إن إجراء هذه الدراسة سيلتزم ببروتوكول IRB المعتمد.

طريقة IRB المعتمدة لمقاربة الموضوعات هي عبر رابط استبيان عبر الإنترنت سيتم توزيعه على جميع المشاركين باستخدام تقنية أخذ عينات كرة الثلج .سيتم نشر رابط الاستبيان والمعلومات على منصات وسائط اجتماعية مختلفة بما في ذلك صفحات Facebook ومجموعات WhatsApp ، حيث سيتم دعوة المشاركين إلى البحث .ستتضمن الدعوة رابط الاستبيان ونموذج الموافقة .الغرض من هذه الدراسة هو استكشاف تأثير COVID-19 على الصحة العقلية ونوعية الحياة (QoL) للسكان البالغين اللبنانيين.

<u>الإجراءات</u>

تدعوك هذه الرسالة إلى:

1. اقرأ وثيقة الموافقة وفكر فيما إذا كنت تريد المشاركة في الدراسة.

وللملاحظة:

- المشاركة طوعية تمامًا.
- سيستغرق إكمال الاستبيان حوالي 20 إلى 30 دقيقة.
- سيتم فقط جمع وتحليل البيانات التي تقدمها في الاستبيان.
- لن يتمكن فريق البحث من الوصول إلى اسمك أو تفاصيل الاتصال بك.
- ستُنشر نتائج الاستطلاع في مقالات بحثية / أطروحة / تقرير مشروع متاحًا مطبوعة من مكتبات الجامعة الأمريكية في بيروت وإلكترونيًا.
- ستكون معايير التضمين هي (1) الرغبة في المشاركة ، (2) الأفراد الذين تزيد أعمارهم عن 18 عامًا والذين لديهم إمكانية الوصول إلى الإنترنت ، و (3) المقيمين في لبنان وقت إجراء المسح.

<u>الفوائد المحتملة للمواطنين و / أو المجتمع</u>

لن تتلقى مدفوعات مقابل المشاركة في هذه الدراسة.

ستساعد نتائج الدراسة في تحديد الفئات الضعيفة المحتملة أو العوامل القابلة للتعديل لتصميم وتقديم برامج تعزيز نفسي محددة للبالغين بهدف تعزيز صحتهم العقلية ورفاههم خلال حقبة.COVID-19

<u>المخاطر المحتملة على الأفراد و / أو المجتمع</u>

مخاطر الدراسة ضئيلة. ولكن نظرًا للتعامل مع الأسئلة الحساسة المحتملة المتعلقة بالصحة العقلية والخوف من COVID-19 ، فهناك قلق محتمل من إثارة المشاعر السلبية ، واستحضار الذكريات التي قد تجدها مرهقة أو مزعجة. في مثل هذه الحالة ، قد تتلقى دعمًا احترافيًا على نفقتك الخاصة عن طريق الاتصال بـ:

• خلال ساعات العمل (8 صباحًا - 5 مساءً): الاتصال بخدمات العيادات الخارجية لقسم الطب النفسي في المركز الطبي للجامعة الأمريكية في بيروت (01-759620 ؛ 01-350000 ، تحويلة 5650) تفيد بأنك شاركت في الاستبيان وطلب استشارة أو طلب التحدث إلى الممرضات.

السرية

ستبقى البيانات التي تم جمعها سرية ومجهولة المصدر.

ستتم مراقبة السجلات وقد يتم تدقيقها من قبل مجلس الهجرة واللاجئين مع ضمان السرية.

المشاركة والانسحاب

إذا وافقت طواعية على المشاركة في هذه الدراسة ، فيمكنك تغيير رأيك و

الانسحاب في أي وقت دون عواقب من أي نوع .لن يترتب على رفض المشاركة أو الانسحاب من الدراسة أي عقوبة أو فقدان المزايا التي يحق للموضوع الحصول عليها بطريقة أخرى ، ولن يؤثر ذلك على علاقتهم بمنظمتهم والجامعة الأمريكية في بيروت / المركز الطبي في الجامعة الأمريكية في بيروت.

أسئلة حول الدراسة

إذا كان لديك أي أسئلة حول الدراسة ، يمكنك التواصل مع الباحث الرئيسي ، الدكتور سامر خَرّوبي, قسم التغذية وعلوم الغذاء، الجامعة الأميركية في بيروت

هاتف: 961-1-350000، تحويلة (4541)، البريد الإلكتروني: sk157@aub.edu.lb

الوصول إلى الاستبيان

إذا قمت بقراءة وثيقة الموافقة والرد على أسئلتك ، فأنت

الموافقة طواعية على المشاركة في الدراسة ؛ يمكنك الوصول إلى الاستطلاع بالنقر فوق

الرابط التالي.

لمزيد من المعلومات والأسئلة حول البحث/ حول حقوقك

مجلس مراجعة مؤسّسي العلوم الإجتماعية والسّلوكية

العنوان: الجامعة الأميركية في بيروت؛ شارع رياض الصّلح، بيروت 2020 1107، لبنان

هاتف: 961-1-374374، تحويلة (5445)، **البريد الإلكتروني**: irb@aub.edu.lb
