# Supplementary material for: The impact of COVID-19 on quality of life among Lebanese adults: a cross-sectional study
Source: Front Public Health. 2025 Jun 18;13:1606720. doi: 10.3389/fpubh.2025.1606720 (PMC12213388; doi:10.3389/fpubh.2025.1606720)
Supplement: Supplementary file 3 [file Data_Sheet_3.pdf]

Appendix 3-Online Survey (English)

I. Sociodemographic Characteristics

1. How old are you? Choose one of the following answers
   - 18-24
   - 25-29
   - 30-39
   - 40-49
   - 50-59
   - 60-69
   - 70+
   - Prefer not to answer
2. Gender: Choose one of the following answers
   - Male
   - Female
   - Prefer not to answer
   - Other: ____________
3. Dwelling region
   - Beirut (capital)
   - Mount Lebanon
   - South Lebanon
   - Bekaa plain
   - North Lebanon
4. Household size
   - Lower than 4 persons
   - 4 persons
   - 5 persons
   - 6 and more

5. Number of rooms
   - < 5 rooms
   - 5 rooms
   - 6 rooms
   - 7 or more
6. What is your primary nationality? Choose one of the following answers
   - Lebanese
   - Prefer not to answer
   - Other:
7. What is the highest level of education you have attained? Choose one of the following answers
   - No schooling completed
   - Below high school, no diploma
   - Some high school, no diploma
   - High school graduate
   - Some college credit, no degree
   - Trade/technical/vocational training
   - Bachelor’s degree

    - Master’s degree
    - Professional degree (ex. M.D., J.D., Pharm.D., M.B.A, etc.)
    - Doctorate degree
    - Prefer not to answer
8. What is your current employment status? Choose one of the following answers
    - Self employed
    - Employed, full time
    - Employed, part time
    - Daily laborer
    - Going to school/university/studying
    - Unemployed, looking for work
    - Unemployed, not looking for work
    - Retired
    - Too ill to work
    - Handicapped, cannot work
    - Housework/ childcare
    - Prefer not to answer
    - Other:______________
9. Work DURING COVID crisis
    - Goes to work now
    - Has absolutely go out
    - Applies social distancing
    - I was licensed from work
    - Job can't be done from home
    - Prefer not to answer
10. Current position AFTER COVID
    - Works on his/her own
    - Owns an enterprise
    - Managerial position
    - Employee
    - Looking for a job
    - Prefer not to answer
11. What is your current marital status? Choose one of the following answers
    - Single
    - Engaged
    - Married
    - Separated
    - Divorced
    - Widowed
    - Prefer not to answer
12. What is your current income level? *Values are according to the official exchange rate 1 USD = 1,515 LBP.* Choose one of the following answers
    - No income
    - Low <675,000LBP (450 USD)
    - Moderate 675,000-1,500,000LBP (450-1,000 USD)
    - Intermediate 1,500,000-3,000,000 LBP (1,000-2,000 USD)

- High income > 3,000,000 LBP (2,000 USD)
- Prefer not to answer

13. Have you or any household member been cut off from your sources of income due to **COVID-19 pandemic**?
    - Completely
    - Partially
    - Not affected

*Please indicate in the next following statements how **COVID-19** in Lebanon may have changed your social and family support.*

14. Getting support from friends
    - Decreased
    - Same as before
    - Increased
15. Getting support from family members
    - Decreased
    - Same as before
    - Increased
16. Shared feeling with family members
    - Decreased
    - Same as before
    - Increased
17. Shared feeling with others when in blue
    - Decreased
    - Same as before
    - Increased
18. Caring for family members' feelings
    - Decreased
    - Same as before
    - Increased

II. Health and Behavioral Characteristics

19. Alcohol consumption
    - Previous
    - None
    - Occasional
    - Regular
20. Cigarette smoking
    - Previous
    - None
    - Occasional
    - Regular
21. Waterpipe smoking
    - Previous

- None
- Occasional
- Regular

22. Violence at home
    - Verbal violence
    - Physical violence
    - Sexual violence
    - Other violence
    - No violence
23. Current Health Coverage
    - No Health coverage
    - Private insurance
    - Social security
    - Other public coverage
24. Do you have a mental illness?
    - Yes
    - No
    - Prefer not to answer
25. Friend diagnosed with mental illness
    - Yes
    - No
    - Prefer not to answer
26. Family member diagnosed with mental illness
    - Yes
    - No
    - Prefer not to answer
27. Do you have chronic illness?
    - Yes
    - No
    - Prefer not to answer
28. Treatment for chronic disease
    - Regular treatment
    - No regular treatment
    - Does not apply
29. Fear no access to treatment
    - No
    - Yes
    - Does not apply
30. Family member has chronic disease
    - No
    - Yes
    - Does not apply
31. Worried family member
    - No
    - Yes
    - Does not apply

32. Exposed to person with COVID-19
    - Yes
    - No
    - Prefer not to answer
33. I have a relative diagnosed with COVID-19
    - Yes
    - No
    - Prefer not to answer
34. I am at risk of being infected with COVID-19
    - Yes
    - No
    - Prefer not to answer
35. I have been quarantined for 14 days
    - Yes
    - No
    - Prefer not to answer
36. Do you follow the COVID news?
    - Yes
    - No
    - Prefer not to answer
37. My main source of information about COVID-19 is
    - Internet
    - TV
    - Friends

III. <u>The fear of COVID-19 scale</u>

	Strongly disagree	Disagree	Neither agree nor disagree	Agree	Strongly agree
I am most afraid of coronavirus-19	1	2	3	4	5
It makes me uncomfortable to think about coronavirus-19	1	2	3	4	5
My hands become clammy when I think about coronavirus-19	1	2	3	4	5
I am afraid of losing my life because of coronavirus-19	1	2	3	4	5
When watching news and stories about coronavirus-19 on social media, I become nervous or anxious	1	2	3	4	5
I cannot sleep because I'm worrying about getting coronavirus-19	1	2	3	4	5
My heart races or palpitates when I think about getting coronavirus-19	1	2	3	4	5

IV. **The Beirut Distress Scale-22 (BDS-22)**

Please rate how often, in the past few weeks, you experienced the following	Not al all	Some of the time	Most of the time	All the time
I feel despaired	0	1	2	3
I think life has no meaning	0	1	2	3
I feel empty	0	1	2	3
I feel on the edge	0	1	2	3
I feel I don’t recognize myself	0	1	2	3
I get angry for ridiculous reasons	0	1	2	3
I isolate myself	0	1	2	3
I lost the desire to learn	0	1	2	3
I lack enthusiasm	0	1	2	3
I don’t know what I want	0	1	2	3
My ideas are puzzled	0	1	2	3
I have constipation or diarrhea	0	1	2	3
I have stomach cramps	0	1	2	3
I have stomach heartburn	0	1	2	3
I find it difficult to relax	0	1	2	3
My mood changes for tiny matters	0	1	2	3
I am in a bad mood	0	1	2	3
I have memory troubles	0	1	2	3
I have difficulty concentrating	0	1	2	3
I don’t know what values to adopt	0	1	2	3
I have panic attacks	0	1	2	3
I worry about little things	0	1	2	3

V. **The Lebanese Anxiety Scale-10 (LAS-10)**

	Not present	Mild	Moderat e	Severe	Very Sever e
I have insomnia (Difficulty in falling asleep, broken sleep, unsatisfying sleep and fatigue on waking, dreams, nightmares, night terrors)	0	1	2	3	4
I have tension (Feelings of tension, fatigability, startle response, moved to tears easily, trembling, feelings of restlessness, inability to relax.)	0	1	2	3	4
I have somatic (muscular) problems (Pains and aches, twitching, stiffness, myoclonic jerks, grinding of teeth, unsteady voice, increased muscular tone)	0	1	2	3	4
I have an anxious mood (Worries, anticipation of the worst, fearful anticipation, irritability)	0	1	2	3	4

I have a depressed mood (Loss of interest, lack of pleasure in hobbies, depression, early waking, diurnal swing).	0	1	2	3	4
I have fears (Of dark, of strangers, of being left alone, of animals, of traffic, of crowds)	0	1	2	3	4
I have intellectual problems (Difficulty in concentration, poor memory)	0	1	2	3	4
I feel inadequate	Never/ Almost Never (1)	Sometimes (2)	Often (3)	Almost Always (4)	
I feel that difficulties are piling up so that I cannot overcome them	Never/ Almost Never (1)	Sometimes (2)	Often (3)	Almost Always (4)	
I feel indecisive	Never/ Almost Never (1)	Sometimes (2)	Often (3)	Almost Always (4)	

VI. COVID-19 – Impact on Quality of Life (COV19-QoL)

Instruction for participants:

Please, choose the number that best represents the degree of your agreement with the statements provided below. Please keep in mind that your estimates reflect your feelings and thoughts during the **past 7 days.**

Due to the spread of the coronavirus,

	**Completely disagree**	**Disagree**	**Neither agree nor disagree**	**Agree**	**Completely Agree**
1. ...I think my quality of life is lower than before	1	2	3	4	5
2. ...I think my mental health has deteriorated	1	2	3	4	5
3. ...I think my physical health may deteriorate	1	2	3	4	5
4. ...I feel more tense than before	1	2	3	4	5
5. ...I feel more depressed than before	1	2	3	4	5
6. ...I feel that my personal safety is at risk	1	2	3	4	5

VII. <u>EQ-5D-5L questions</u>

Under each heading, please tick the ONE box that best describes your health TODAY.

MOBILITY

I have no problems in walking about ❑
I have slight problems in walking about ❑
I have moderate problems in walking about ❑
I have severe problems in walking about ❑
I am unable to walk about ❑

SELF-CARE

I have no problems washing or dressing myself ❑
I have slight problems washing or dressing myself ❑
I have moderate problems washing or dressing myself ❑
I have severe problems washing or dressing myself ❑
I am unable to wash or dress myself ❑

USUAL ACTIVITIES *(e.g. work, study, housework, family or leisure activities)*

I have no problems doing my usual activities ❑
I have slight problems doing my usual activities ❑
I have moderate problems doing my usual activities ❑
I have severe problems doing my usual activities ❑
I am unable to do my usual activities ❑

PAIN / DISCOMFORT

I have no pain or discomfort ❑
I have slight pain or discomfort ❑
I have moderate pain or discomfort ❑
I have severe pain or discomfort ❑
I have extreme pain or discomfort ❑

ANXIETY / DEPRESSION

I am not anxious or depressed ❑
I am slightly anxious or depressed ❑
I am moderately anxious or depressed ❑
I am severely anxious or depressed ❑
I am extremely anxious or depressed ❑

- We would like to know how good or bad your health is TODAY.
- This scale is numbered from 0 to 100.
- 100 means the best health you can imagine.
- 0 means the worst health you can imagine.
- Mark an X on the scale to indicate how your health is TODAY.
- Now, please write the number you marked on the scale in the box below.

YOUR HEALTH TODAY =

The best health
you can imagine

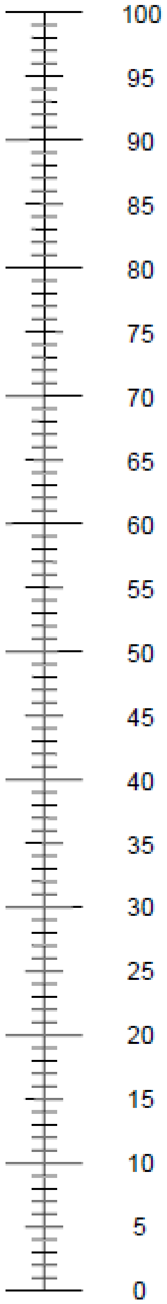

The worst health
you can imagine

Appendix 3-Online Survey (Arabic)

استطلاع على الانترنت

تأثير جائحة COVID-19 على الصحة النفسية وجودة الحياة بين اللبنانيين: دراسة مقطعية

I. الخصائص الاجتماعية الديموغرافية

1. كم عمرك؟ اختر واحدة من الإجابات التالية
   - 18-24
   - 25-29
   - 30-39
   - 40-49
   - 50-59
   - 60-69
   - 70+
   - افضل عدم الاجابة
2. الجنس :اختر إحدى الإجابات التالية
   - الذكر
   - أنثى
   - افضل عدم الاجابة
   - آخر:___________
3. منطقة المسكن
   - بيروت
   - جبل لبنان
   - جنوب لبنان
   - سهل البقاع
   - شمال لبنان
4. حجم الأسرة
   - أقل من 4 أشخاص
   - 4اشخاص
   - 5أشخاص
   - 6وأكثر
5. عدد الغرف
   - 5<غرف
   - 5غرف
   - 6غرف
   - 7أو أكثر
6. ما هي جنسيتك الأساسية؟ اختر واحدة من الإجابات التالية
   - لبناني
   - افضل عدم الاجابة
   - آخر:
7. ما هو أعلى مستوى تعليمي وصلت إليه؟ اختر واحدة من الإجابات التالية
   - لم يكتمل التعليم
   - تحت المدرسة الثانوية ، لا دبلوم

- بعض المدارس الثانوية ، لا دبلوم
- خريج الثانوية
- بعض الائتمان الجامعي ، بدون شهادة
- التجارة / التدريب الفني / المهني
- درجة البكالريوس
- ماجيستير
- الدرجة المهنية مثل MD ، JD ، Pharm.D. ، MBA ، إلخ
- درجة الدكتوراه
- افضل عدم الاجابة

8. ماهو وضعك الوظيفي حاليا؟ اختر واحدة من الإجابات التالية
   - العاملون لحسابهم الخاص
   - موظف بدوام كامل
   - يعمل بدوام جزئي
   - عامل يومي
   - الذهاب إلى المدرسة / الجامعة / الدراسة
   - عاطل عن العمل ، يبحث عن عمل
   - عاطل عن العمل ، لا يبحث عن عمل
   - متقاعد
   - مريض جدا للعمل
   - معاق ، لا يمكن أن يعمل
   - الأعمال المنزلية / رعاية الأطفال
   - افضل عدم الاجابة
   - آخر_____________:
9. العمل أثناء أزمةCOVID
   - يذهب للعمل الآن
   - بالتأكيد خرج
   - يطبق التباعد الاجتماعي
   - لقد حصلت على ترخيص من العمل
   - لا يمكن أن يتم العمل من المنزل
   - افضل عدم الاجابة
10. الموقف الحالي بعدCOVID
    - يعمل بمفرده
    - يمتلك مؤسسة
    - منصب إداري
    - موظف
    - تبحث عن وظيفة
    - افضل عدم الاجابة
11. ما هو الوضع العائلي الحالي؟ اختر واحدة من الإجابات التالية
    - غير مرتبطة
    - مخطوب \ مخطوبة
    - متزوج
    - فصل

- مطلقة
- الأرامل
- افضل عدم الاجابة

12. ما هو مستوى دخلك الحالي؟ القيم طبقًا لسعر الصرف الرسمي 1 دولار أمريكي 1515 = ليرة لبنانية .اختر واحدة من الإجابات التالية
    - لا دخل
    - منخفضة < 675,000 ليرة لبنانية (450 دولارًا)أمريكيًا
    - متوسطة 675,000-1,500,000LBP (450-1,000) دولار أمريكي
    - متوسط 1,500,000-3,000,000 LBP (1,000-2,000 دولار أمريكي)
    - ذات الدخل المرتفع > 3,000,000 LBP (2,000 دولار أمريكي)
    - افضل عدم الاجابة
13. هل تم قطعك أنت أو أي فرد من أفراد أسرتك عن مصادر دخلك بسبب جائحةCOVID-19 ؟
    - تماما
    - جزئيا
    - لم تتأثر

يرجى الإشارة في البيانات التالية إلى كيف أن فيروس كورونا في لبنان قد غيّر الدعم الاجتماعي والعائلي.

14. الحصول على الدعم من الأصدقاء
    - انخفضت
    - نفسه كما كان من قبل
    - زاد
15. الحصول على الدعم من أفراد الأسرة
    - انخفضت
    - نفسه كما كان من قبل
    - زاد
16. تقاسم الشعور مع أفراد الأسرة
    - انخفضت
    - نفسه كما كان من قبل
    - زاد
17. تقاسم الشعور مع الآخرين عندما يرتدون الأزرق
    - انخفضت
    - نفسه كما كان من قبل
    - زاد
18. الاهتمام بمشاعر أفراد الأسرة
    - انخفضت
    - نفسه كما كان من قبل
    - زاد

II. الخصائص الصحية والسلوكية

19. استهلاك الكحول
    - السابق

- لا شيء
- من حين لآخر
- منتظم

20. تدخين السجائر
    - السابق
    - لا شيء
    - من حين لآخر
    - منتظم
21. تدخين الشيشة
    - السابق
    - لا شيء
    - من حين لآخر
    - منتظم
22. العنف في المنزل
    - العنف اللفظي
    - عنف جسدي
    - العنف الجنسي
    - أعمال عنف أخرى
    - لا عنف
23. التغطية الصحية الحالية
    - لا توجد تغطية صحية
    - التأمين الخاص
    - الضمان الاجتماعي
    - تغطية عامة أخرى
24. هل لديك مرض عقلي؟
    - نعم
    - لا
    - افضل عدم الاجابة
25. صديق تم تشخيصه بمرض عقلي
    - نعم
    - لا
    - افضل عدم الاجابة
26. تم تشخيص أحد أفراد الأسرة بمرض عقلي
    - نعم
    - لا
    - افضل عدم الاجابة
27. هل لديك مرض مزمن؟
    - نعم
    - لا
    - افضل عدم الاجابة
28. علاج الأمراض المزمنة
    - العلاج المنتظم

- لا يوجد علاج منتظم
- لا ينطبق

29. لا تخف من الوصول إلى العلاج
   - لا
   - نعم
   - لا ينطبق
30. أحد أفراد الأسرة مصاب بمرض مزمن
   - لا
   - نعم
   - لا ينطبق
31. أحد أفراد الأسرة القلق
   - لا
   - نعم
   - لا ينطبق
32. معرضة لشخص مصاب بـCOVID-19
   - نعم
   - لا
   - افضل عدم الاجابة
33. لدي قريب تم تشخيصه بـCOVID-19
   - نعم
   - لا
   - افضل عدم الاجابة
34. أنا معرض لخطر الإصابة بـCOVID-19
   - نعم
   - لا
   - افضل عدم الاجابة
35. لقد تم الحجر الصحي لي لمدة 14 يومًا
   - نعم
   - لا
   - افضل عدم الاجابة
36. هل تتابع أخبارCOVID ؟
   - نعم
   - لا
   - افضل عدم الاجابة
37. المصدر الرئيسي لمعلوماتي حول COVID-19 هو
   - الإنترنت
   - تلفزيون
   - اصحاب

III. الخوف من انتشار مرض19- COVID

	أعارض بشدة	تعارض	لا أوافق ولا أرفض	يوافق على	موافق بشدة
أنا أكثر خوفًا من فيروس كورونا19	1	2	3	4	5
أشعر بعدم الارتياح عندما أفكر في فيروس كورونا19-	1	2	3	4	5
تصبح يدي رطبة عندما أفكر في فيروس كورونا19-	1	2	3	4	5
أخشى أن أفقد حياتي بسبب فيروس كورونا19	1	2	3	4	5
عند مشاهدة الأخبار والقصص حول فيروس كورونا 19- على وسائل التواصل الاجتماعي ، أشعر بالتوتر أو القلق	1	2	3	4	5
لا أستطيع النوم لأنني قلق من الإصابة بفيروس كورونا19	1	2	3	4	5
قلبي يتسارع أو يخفق عندما أفكر في الإصابة بفيروس كورونا19-	1	2	3	4	5

IV. مقياس الاستغاثة في بيروت-22 (BDS-22)

يُرجى تقييم عدد المرات التي واجهت فيها ما يلي في الأسابيع القليلة الماضية	لا الله جميع	بعض من الوقت	معظم الوقت	طوال الوقت
أشعر باليأس	0	1	2	3
أعتقد أن الحياة ليس لها معنى	0	1	2	3
أشعر بالفراغ	0	1	2	3
أشعر بأنني على حافة الهاوية	0	1	2	3
أشعر أنني لا أعرف نفسي	0	1	2	3
أنا أغضب لأسباب سخيفة	0	1	2	3
أنا أعزل نفسي	0	1	2	3
لقد فقدت الرغبة في التعلم	0	1	2	3
أنا أفتقر إلى الحماس	0	1	2	3
لا اعرف ماذا اريد	0	1	2	3
أفكاري في حيرة	0	1	2	3
أعاني من إمساك أو إسهال	0	1	2	3
أعاني من تقلصات في المعدة	0	1	2	3
أعاني من حرقة في المعدة	0	1	2	3
أجد صعوبة في الاسترخاء	0	1	2	3
يتغير مزاجي للأمور الصغيرة	0	1	2	3
انا في مزا ج سيء	0	1	2	3
لدي مشاكل في الذاكرة	0	1	2	3
أجد صعوبة في التركيز	0	1	2	3
لا أعرف ما هي القيم التي يجب تبنيها	0	1	2	3
لدي نوبات هلع	0	1	2	3
أنا قلق بشأن الأشياء الصغيرة	0	1	2	3

V. **مقياس القلق اللبناني -10 (LAS-10)**

	ليس<br>حاضر	خفيف	معتدل	شديدة	قاسيه جدا
أعاني من الأرق) صعوبة النوم ، النوم المتقطع ، النوم غير المرضي ، التعب عند الاستيقاظ ، الأحلام ، الكوابيس ، الذعر الليلي)	0	1	2	3	4
لدي توتر(مشاعر توتر ، تعب ، ذهول استجابة ، انتقلت إلى البكاء بسهولة ، يرتجف ، مشاعر الأرق وعدم القدرة على الاسترخاء).	0	1	2	3	4
أعاني من مشاكل جسدية(عضلية)<br>(آلام وآلام ، ارتعاش ، تصلب ، رمع عضلي ، صرير الأسنان ، صوت غير مستقر ، زيادة نغمة عضلية)	0	1	2	3	4
لدي مزاج قلق<br>(القلق ، توقع الأسوأ ، الترقب المخيف ، التهيج)	0	1	2	3	4
لدي مزاج مكتئب<br>(فقدان الاهتمام ، قلة المتعة بالهوايات ، الاكتئاب ، الاستيقاظ المبكر ، التأرجح النهاري)	0	1	2	3	4
لدي مخاوف<br>(من الظلام ، من الغرباء ، من ترك وحيدا ، من الحيوانات ، من حركة المرور ، من الحشود)	0	1	2	3	4
لدي مشاكل فكرية<br>(صعوبة في التركيز وضعف الذاكرة)	0	1	2	3	4
أشعر بعدم كفاية	أبدا / تقريبا أبدا(1)	أحيانا(2)	في كثير من الأحيان(3)	تقريبا دائما (4)	
أشعر أن الصعوبات تتراكم حتى لا أستطيع التغلب عليها	أبدا / تقريبا أبدا(1)	أحيانا(2)	في كثير من الأحيان(3)	تقريبا دائما (4)	
أشعر بالتردد	أبدا / تقريبا أبدا(1)	أحيانا(2)	في كثير من الأحيان(3)	تقريبا دائما (4)	

VI. التأثير- ***COVID-19*** على جودة الحياة (COV19-QoL)

تعليمات للمشاركين:

من فضلك ، اختر الرقم الذي يمثل على أفضل وجه درجة موافقتك على البيانات الواردة أدناه .يرجى أن تضع في اعتبارك أن تقديراتك تعكس مشاعرك وأفكارك خلال الأيام السبعة الماضية.

**أتفق تماما**	**يوافق على**	**لا توافق ولا تعارض**	**تعارض**	**تماما تعارض**	بسبب انتشار فيروس كورونا ،
5	4	3	2	1	1.أعتقد أن نوعية حياتي أقل من ذي قبل
5	4	3	2	1	2. أعتقد أن صحتي العقلية قد تدهورت
5	4	3	2	1	3.أعتقد أن صحتي الجسدية قد تتدهور
5	4	3	2	1	4.أشعر بتوتر أكثر من ذي قبل
5	4	3	2	1	5.أشعر بالاكتئاب أكثر من ذي قبل
5	4	3	2	1	6.أشعر أن سلامتي الشخصية في خطر

VII. **استبيان صحّي EQ5D-النسخة العربية للبنان**

تحت كل عنوان مما يلي، ضع علامة في مربع واحد فقط عند أفضل عبارة تصف حالتك الصحية **اليوم**.

القدرة على الحركة

ليس لدي أي مشاكل أثناء المشي ❑

أعاني مشاكل طفيفة أثناء المشي ❑

أعاني مشاكلَ متوسطة أثناء المشي ❑

عاني مشاكلَ حا دة أثناء المشي ❑

ليس لدي القدرة على المشي ❑

العناية الشخصية

ليس لدي أي مشاكل في الاستحمام أو ارتداء ملابسي بنفسي ❑

أعاني مشاكلَ طفيفة عند الاستحمام أو ارتداء ملابسي بنفسي ❑

أعاني مشاكلَ متوسطة عند الاستحمام أو ارتداء ملابسي بنفسي ❑

أعاني مشاكلَ حا دة عند الاستحمام أو ارتداء ملابسي بنفسي ❑

ليس لدي القدرة على الاستحمام أو ارتداء الملابس بنفسي ❑

الأنشطة المعتادة *(مثل العمل، الدراسة، الأعمال المنزلية، الأنشطة الأسرية أو الترفيهية)*

ليس لدي أي مشاكل في ممارسة نشاطاتي المعتادة ❑

أعاني مشاكلَ طفيفة في القيام بنشاطاتي المعتادة ❑

أعاني مشاكلَ متوسطة في ممارسة نشاطاتي المعتادة ❑

أعاني مشاكلَ حا دة في ممارسة نشاطاتي المعتادة ❑

ليس لديّ القدرة على ممارسة نشاطاتي المعتادة ❑

الألم / الإحساس بعدم الراحة

- ليس لدي أي ألم أو انزعاج ❑
- أعاني ألما طفيفا أو انزعاجا طفيفاً ❑
- أعاني ألما متوسطا أو انزعاجا متوسط ❑
- أعاني ألما حا داً أو انزعاجا حا د اً ❑
- أعاني ألما شديداً جداً أو انزعاجا شديداً جد اً ❑

القلق / الاكتئاب

- لا أعاني أ ي قلق أو اكتئاب ❑
- أعاني قلقا طفيفا أو اكتئابا طفيف ❑
- أعاني قلقا متوسطا أو اكتئابا متوسطاً ❑
- أعاني قلقا حا داً أو اكتئابا حا د اً ❑
- أعاني قلقا شديداً جداً أو اكتئابا شديداً جدا ❑

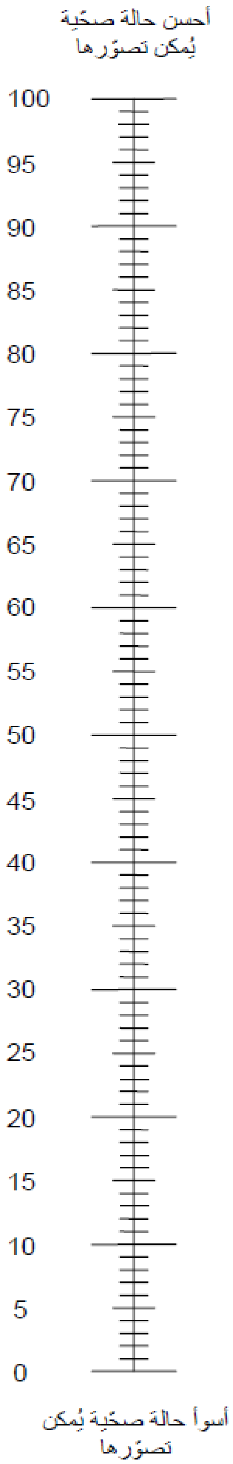

- نود أن نعرف مدى سوء حالتك ال صحية أو سلامتها اليوم.
- هذا المقياس مدرج من الرقم 0 حتى 100
- الرقم 100 يعني أحسن حالة صحية يمكنك تصورها
- 0 يعني أسوأ حالة صحية يمكنك تصورها.
- ضع X على المقياس للإشارة إلى حالتك الصحية اليوم.
- الآن، قم رجاء بكتابة الرقم الذي أشرت إليه على المقياس في المربع أدناه.

حالتك الصحية اليوم = ☐
